# A lactylation-related transcriptional signature as candidate biomarkers for the early detection of polycystic ovary syndrome: an integrated genomic analysis

**DOI:** 10.1186/s13048-026-02208-5

**Published:** 2026-07-23

**Authors:** Rui Wei, Hao Zhang, Wenting Xu, Changjiang Qin

**Affiliations:** 1 Translational Medical Center, The Second Clinical Medical College of Henan University, Kaifeng, 475000 China; 2 Department of General Surgery, The Second Clinical Medical College of Henan University, Kaifeng, 475000 China; 3 Quality Control Office, The People’s Hospital of Kaifeng, Kaifeng, 475000 China

**Keywords:** Polycystic ovary syndrome, Lactylation-related Transcriptional signatures, Early detection, Candidate biomarkers, Integrated genomic analysis

## Abstract

**Background:**

Polycystic Ovary Syndrome (PCOS) is a prevalent endocrine disorder that negatively impacts women’s reproductive and metabolic health, leading to complications such as irregular menstruation, infertility, obesity, and metabolic syndrome. Current diagnostic methods, which primarily rely on clinical symptoms and hormone levels, lack specificity in early detection. This study aimed to identify lactylation-related transcriptional signatures as candidate biomarkers for the early detection of PCOS.

**Methods:**

We conducted a comprehensive bioinformatic analysis using transcriptomic data from granulosa cells of PCOS patients in the GEO database. Patients were categorized into two molecular subtypes via consensus clustering based on the expression of lactylation-related genes. Subsequently, diagnostic models were constructed and validated to evaluate the potential of these transcriptional signatures as early diagnostic markers. External validation was performed using an independent dataset, and selected candidate genes were further examined in DHEA-treated KGN cells.

**Results:**

We identified nine key genes (HK3, SDC3, TGFBI, ZYX, LSP1, HMGA1, LCP1, B3GAT1, and ZNF280C) that were significantly differentially expressed in PCOS granulosa cell transcriptomic datasets. Pathway enrichment analyses revealed their involvement in energy metabolism, cell proliferation and apoptosis, which highlights their potential roles in PCOS pathogenesis. A random forest-based diagnostic model showed high internal predictive performance, with an ROC AUC of 0.993; however, external validation yielded a lower AUC of 0.628, indicating limited generalizability and the need for further validation.

**Conclusion:**

These findings suggest that lactylation-related transcriptional signatures may serve as potential candidate biomarkers for the early detection of PCOS. However, because this study was based primarily on transcriptomic inference and did not directly measure lactylation modifications, the results should be interpreted as hypothesis-generating. Future studies should validate these biomarkers in larger clinical cohorts, primary granulosa cells, and through direct lactylation or proteomic assays.

**Supplementary Information:**

The online version contains supplementary material available at https://doi.org/10.1186/s13048-026-02208-5.

## Background

Polycystic Ovary Syndrome (PCOS) is a common endocrine disorder affecting approximately 10–13% of women worldwide, with a complex etiology involving genetic, environmental, and metabolic factors [1]. Its clinical manifestations include irregular menstruation, hyperandrogenism, and polycystic ovaries. These symptoms not only impair women’s fertility but are also closely related to metabolic syndrome, cardiovascular diseases, and mental health issues [2]. Current diagnostic criteria for PCOS, based on clinical symptoms and imaging, often result in delayed diagnosis and treatment, indicating the need for reliable biomarkers for early detection [3]. The heterogeneity of PCOS, along with traditional biomarkers’ lack of specificity and sensitivity [4], complicates diagnosis, necessitating the integration of novel molecular insights related to metabolic dysregulation for improved understanding of the disease [5].

Recent advances in genomics and bioinformatics have opened new avenues for exploring the role of post-translational modifications in PCOS pathogenesis. It is important to note that prior transcriptomic and systems biology studies have already established the significance of dysregulated gene signatures, immune-metabolic pathways, and hub gene networks in PCOS and related reproductive disorders [6–8]. Building upon this foundation, we turned our focus to lactylation, a novel post-translational modification involving the covalent addition of lactate to lysine residues on proteins, has emerged as a potential regulator of cellular metabolism and inflammation, both central to PCOS pathophysiology. The identification of lactylation-related genes (LRGs) signatures may provide candidate biomarkers for early detection, offering insights into the underlying metabolic disturbances associated with PCOS [9, 10]. Studies indicate that lactylation may be closely related to the metabolic abnormalities of PCOS, especially insulin resistance and hyperandrogenism. Specifically, the accumulation of lactate might affect ovarian function and hormone secretion by triggering lactylation, thus exacerbating the symptoms of PCOS [11]. Furthermore, the role of lactylation in regulating cellular metabolism and gene expression may provide new insights into the underlying mechanisms of PCOS [12]. In PCOS patients, increased lactylation may be associated with metabolic reprogramming in the ovaries. Research shows that lactylation modulates glycolytic pathways by altering the activity of key metabolic enzymes implicated in PCOS pathogenesis [13]. For instance, lactylation-induced modifications of specific enzymes may reduce their activity, thereby affecting ovarian hormone synthesis and follicular development [14]. Thus, the genes involved in the lactylation process may serve not only as candidate biomarkers but also as potential therapeutic targets in PCOS. Beyond metabolism, lactylation is implicated in immune regulation: it may drive macrophages toward a pro-inflammatory phenotype, potentially fueling the chronic low-grade inflammation associated with PCOS [15, 16]. Given these multifaceted roles, exploring lactylation-related genes represents a promising avenue for identifying both candidate biomarkers and potential therapeutic targets for PCOS [17].

To address the current research gap, this study aimed to identify a lactylation-related transcriptional signature as candidate biomarkers for the early detection of PCOS. We conducted a comprehensive analysis of granulosa cell RNA-seq data from PCOS patients in the GEO database. By integrating differential expression analysis with WGCNA, we identified hub LRGs. Unsupervised consensus clustering was then used to stratify patients into molecular subtypes based on the expression patterns of these genes, exploring their association with PCOS heterogeneity. Furthermore, we developed and validated a diagnostic model based on this transcriptional signature to assess their clinical utility [18]. By combining molecular biology with computational approaches, this research seeks to uncover potential diagnostic tools that could contribute to the improved clinical management of PCOS and patient outcomes.

## Methods

### Data acquirement

Data were obtained from the granulosa cells of PCOS patients in the GEO database (https://www.ncbi.nlm.nih.gov/geo/). Two datasets, GSE34526 and GSE173160, were selected for further analysis. GSE34526 was used as the test dataset, while GSE173160 served as the validation dataset. Detailed information about the datasets is presented in Table 1. The lactylation-related genes were collected from previous research [19] and the MsigDB (https://www.gsea-msigdb.org/gsea/msigdb/) [20]. After removing duplicates, a total of 658 unique genes were identified and listed in Supplementary Table S1. The LRG set was compiled to represent transcriptional programs associated with lactylation, including genes involved in lactate metabolism, glycolytic activity, and previously reported lactylation-related regulatory processes. It is crucial to note that, as this study utilized transcriptomic data, the LRG set serves as a proxy for lactylation-related transcriptional activity and does not constitute direct evidence of lysine lactylation modification or enzymatic activity.

**Table 1:** Information of GSE datasets

Datasets	Platform	Species	Data type	Total sample	Control sample	PCOS
GSE34526	GPL570	Homo sapiens	Microarray	10	3	7
GSE173160	GPL24676	Homo sapiens	RNA-seq	12	6	6

### DEGs identification and functional annotation

We used the ‘limma’ R package (version 3.62.2) for data normalization and differential expression analysis to identify DEGs in patients with PCOS compared with healthy controls [21]. We established significance criteria for identifying differentially expressed genes (DEGs) as |log_2_FC| > 2 and *p*-value < 0.05. We visualized the DEGs using the following R packages: ComplexHeatmap (version 2.13.1), dplyr (version 1.1.4), ggplot2 (version 3.5.2), and ggrepel (version 0.9.6) [22]. To elucidate the functional differences between patients with PCOS and healthy controls, we used the ‘clusterProfiler’ package (version 4.4.4) to performed Gene Ontology (GO) enrichment analysis and KEGG pathway analysis on the DEGs [23].

### Immune cells infiltration analysis

The CIBERSORTx website (https://cibersortx.stanford.edu/) was used to obtain the signature matrix and gene expression profiles for the 22 immune cell types, based on the CIBERSORT algorithm (version 1.03). This method was applied to calculate the immune cell infiltration for each sample. The immune cell infiltration results were visualized using the ‘ggplot2’ package (version 3.5.2) [24].

### Weighted Gene Co–Expression Network Analysis (WGCNA)

WGCNA is a sophisticated systems biology method for elucidating patterns of association between genes in microarray gene expression data. The method helps to identify sets of genes with strong co-variation and reveal potential candidate biomarker genes or therapeutic targets by exploring the intrinsic linkages within the set of genes and their correlation with the phenotype [25]. In this study, the top 20% of genes with the largest variance were selected from the normalized mRNA expression data for WGCNA, and Pearson correlation coefficients were computed to construct an adjacency matrix. The soft-threshold power for this matrix was set to 4, followed by transformation into a Topological Overlap Matrix (TOM). Gene modules with similar expression patterns were identified using a dynamic tree–cutting method. Genes with a correlation coefficient > 0.5 in the module exhibiting the strongest association with PCOS were selected as characteristic genes associated with PCOS. These genes were intersected with both DEGs and LRGs to identify LRGs significantly associated with PCOS, also known as hub genes.

### Gene Set Enrichment Analysis (GSEA)

GSEA was conducted by first performing differential expression analysis using the ‘limma’ package (version 3.62.2), followed by functional annotation with ‘org.Hs.eg.db’ (version 3.20.0) and ‘clusterProfiler’ (version 4.14.6) packages to identify signaling pathways associated with the hub genes across distinct expression patterns [26]. Gene sets with an adjusted *p*-value < 0.05 were considered statistically significant. From these, the top three enriched pathways in both high- and low-expression cohorts-ranked by absolute Normalized Enrichment Score (|NES|), were selected for further visualization. The ‘enrichplot’ (version 1.26.6) package was used for the visualization of enrichment results.

### Transcriptional regulatory network analysis of Hub genes

Putative transcription factors (TFs) regulating hub genes were identified using JASPAR (https://jaspar.elixir.no/about/), a curated repository of transcription factor binding profiles characterized by position weight matrices. Protein-protein interaction (PPI) networks were subsequently constructed using the STRING database (https://string-db.org) with high-confidence interaction thresholds (combined score > 0.7). Network topology was visualized using the ‘igraph’ (v1.4.1) and ‘ggraph’ (v2.1.0) packages in R, employing force-directed layout algorithms to optimize node spatial representation [27].

### Subtypes and functional characterization of PCOS

We used expression profiles of PCOS-associated LRGs to establish subtypes through unsupervised consensus clustering. Consensus clustering was performed using the ‘ConsensusClusterPlus’ package (version 1.70.0) [28]. To ensure clustering robustness, 1000 iterations were conducted, with the sample consistency cutoff set at 0.8 and feature consistency cutoff at 1; hierarchical clustering was used as the clustering method. Differences in the immune microenvironment were assessed, and Gene Set Variation Analysis (GSVA) was conducted between the two subtypes [29]. Differential expression analysis was performed using the ‘limma’ package (v3.62.2) with thresholds of |log_2_FC| > 1 and FDR < 0.05, followed by functional annotation of subtype–discriminatory genes via GO and KEGG enrichment.

### Diagnostic model construction and validation

Random Forest (RF), Support Vector Machine (SVM), Generalized Linear Model (GLM), Logistic Regression (LR), Naive Bayes (NB) and K-Nearest Neighbors (KNN) were employed to develop predictive models for identifying PCOS-associated genes within the LRG set [30]. The GSE34526 dataset was used as the discovery dataset for model development and was randomly divided into a 70% training set and a 30% testing set. Model performance was evaluated using residual boxplots, reverse cumulative distribution of residuals, receiver operating characteristic (ROC) curves, calibration curves, and decision curve analysis (DCA). The algorithm with the best overall internal performance was selected as the optimal model. Given the small sample size of GSE34526, the machine-learning analysis was considered exploratory. A fully nested cross-validation framework was not feasible, and feature selection was not repeated within each cross-validation fold. Therefore, the possibility of feature-selection bias and overfitting could not be completely excluded. To partially assess model generalizability, the selected model was further validated in an independent external dataset, GSE173160. The discrepancy between internal and external performance was interpreted cautiously.

### KGN cell culture and dehydroepiandrosterone (DHEA) treatment

KGN human granulosa-like tumor cell line was purchased from Hefei Wanwu Biotechnology Co., Ltd. (Hefei, China). Cells were cultured in DMEM/F12 medium (Sigma-Aldrich, D6421) supplemented with 10% fetal bovine serum (FBS; Gibco, 10270–106) and 1% penicillin-streptomycin (Sigma-Aldrich, P4333) at 37 °C in a humidified 5% CO₂ atmosphere. DHEA (MedChemExpress, HY–14650) was dissolved in dimethyl sulfoxide (DMSO) to prepare a 1 mM stock solution, which was subsequently diluted in culture medium to a final working concentration of 10 µM. Following 24 h of exposure to DHEA, cells were harvested for downstream analyses.

### Quantitative real–time reverse transcription polymerase chain reaction (qRT-PCR)

Total RNA was extracted using TRNzol Universal Reagent (Tiangen Biotech, DP405) in accordance with the manufacturer’s protocol. Reverse transcription was performed with 1 µg of total RNA using the SweScript All-in-One RT-PCR Kit (Servicebio, G3335). Quantitative PCR amplifications were performed in triplicate using 2× Fast SYBR Green qPCR Master Mix (Servicebio, G3323) on a QuantStudio 5 Real-Time PCR System (Applied Biosystems). Primer sequences for the target genes are listed in Supplementary Table S2, with amplification efficiency validated by standard curve analysis. GAPDH expression was used as the endogenous control for data normalization, and relative gene expression levels were calculated using the 2^(-ΔΔCt) method.

### Statistical analysis

All experiments were performed independently at least three times. Data are presented as mean ± standard deviation (SD). Statistical analyses were conducted using GraphPad Prism 9.0 software (GraphPad Inc.). Two-group comparisons were analyzed with Student’s t-test, while multiple group comparisons were assessed using one-way ANOVA followed by Tukey’s post–hoc test. *p*-value < 0.05 was considered statistically significant.

## Results

### Expression of DEGs and functional enrichment analyses in PCOS

**Fig. 1:**
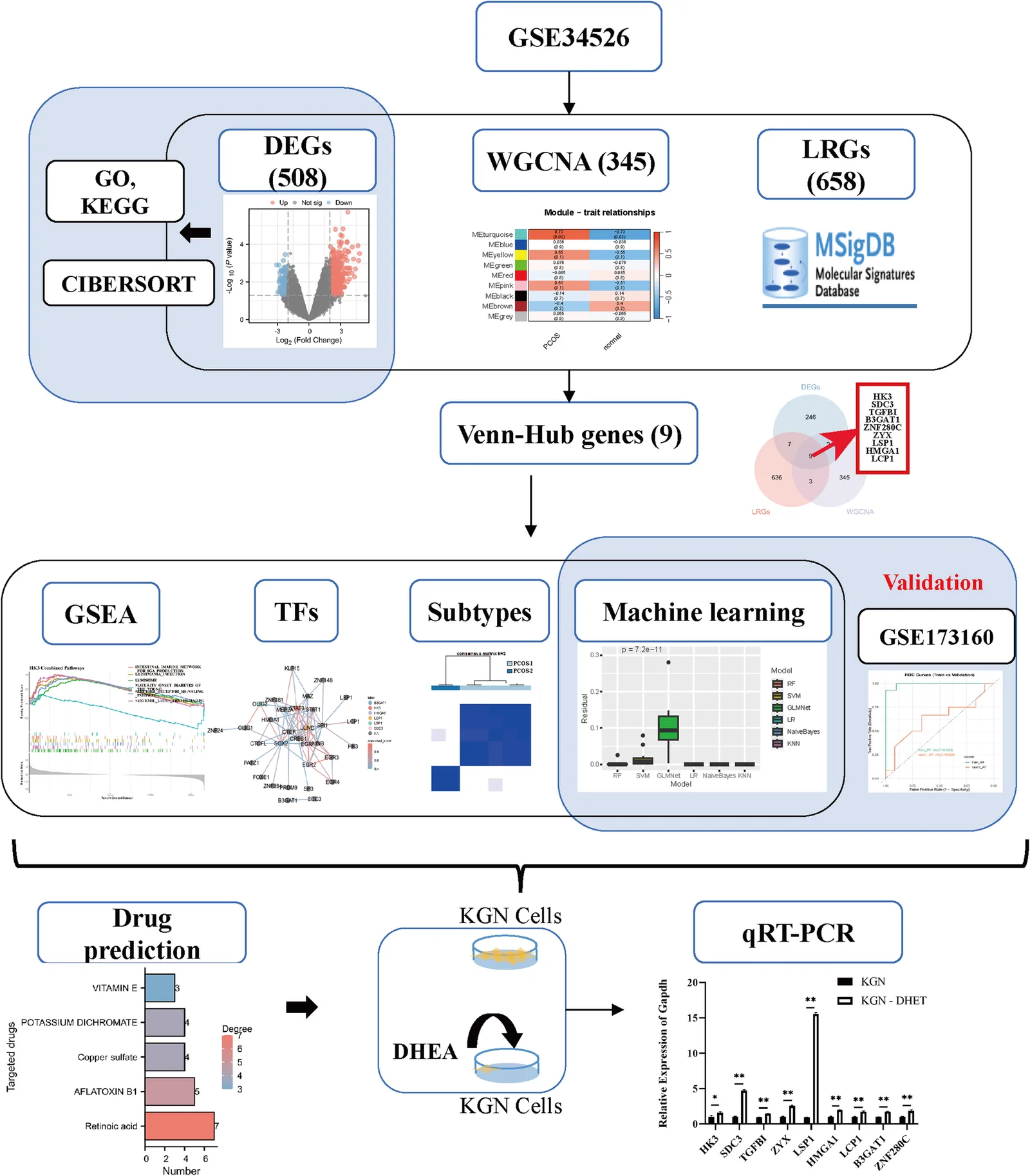
Workflow flowchart

**Fig. 2:**
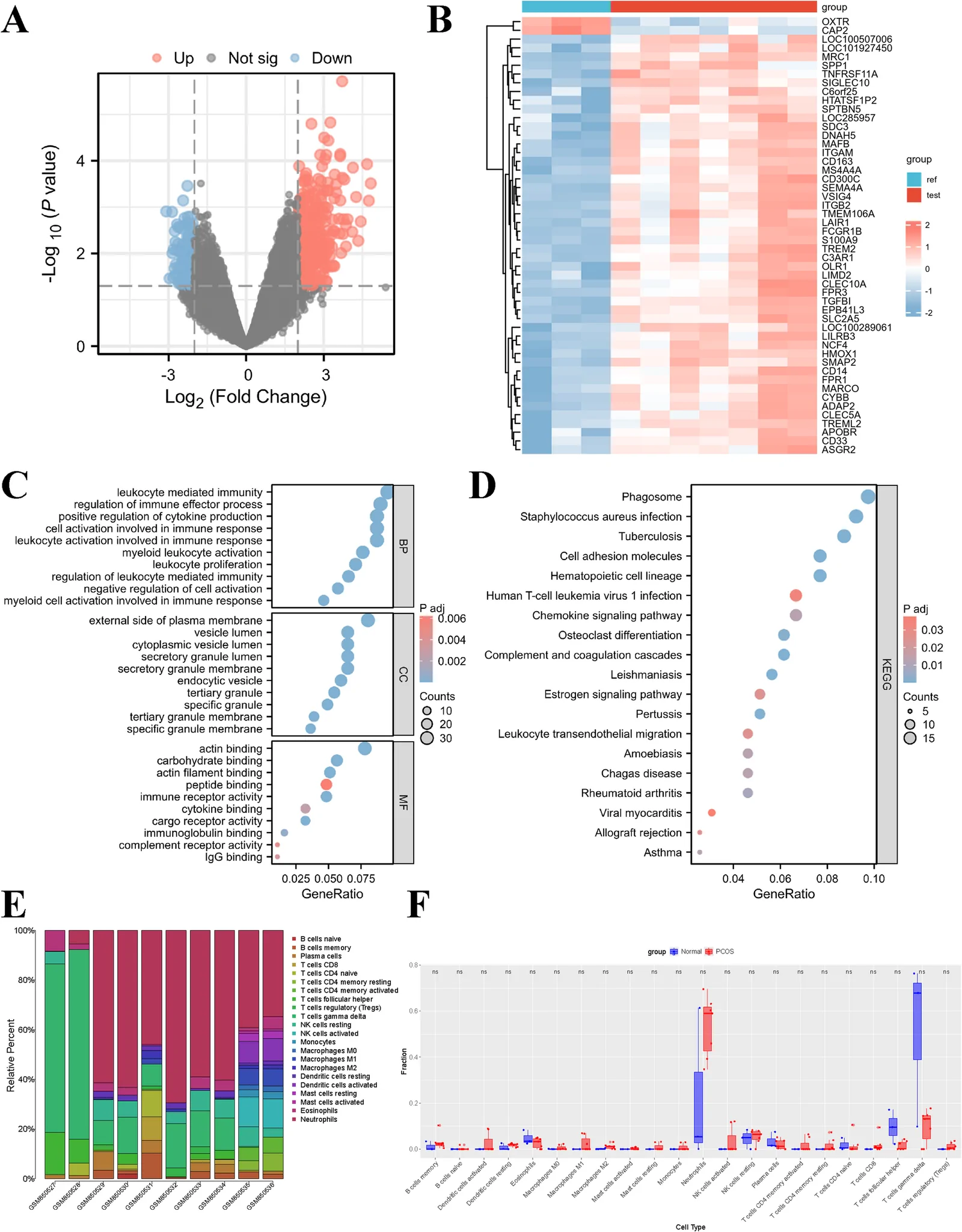
Gene expression profiles and immune cell infiltration patterns in PCOS. **A** Volcano plot of DEGs. **B** Heatmap showing expression patterns of the top 30 upregulated and top 30 downregulated genes. **C** Top 10 biological processes, cellular components, and molecular functions identified by GO analysis. **D** Top 30 pathways identified in KEGG analysis. **E** Immune cell infiltration landscape across samples from the GSE34526 dataset. **F** Comparisons of immune cell infiltration between healthy controls and PCOS patients (ns, not significant; *p < 0.05; **p < 0.01; ***p < 0.001)

Figure 1 illustrates the overall workflow of this study. Differential expression analysis of the GSE34526 dataset using the ‘limma’ package identified a total of 508 DEGs between the PCOS and control groups (|log_2_FC| > 2, *p* < 0.05), including 377 upregulated and 131 downregulated genes (Fig. 2A). The heatmap in Fig. 2B displays the expression patterns of the top 30 upregulated and downregulated genes. The volcano plot confirms a distinct distribution of these genes between the PCOS and control groups. Following ID conversion, 433 DEGs (85.2%) were retained for subsequent functional analyses. Both GO and KEGG enrichment analyses of these 433 DEGs revealed a pronounced focus on the immune response in PCOS. GO enrichment analysis highlighted significant enrichment in biological processes such as leukocyte mediated immunity, regulation of immune effector process, cell activation involved in immune response, and myeloid cell activation involved in immune response (Fig. 2C). KEGG pathway analysis further corroborated this finding, showing significant enrichment in pathways including Phagosome, Staphylococcus aureus infection, chemokine signaling pathway, and Estrogen signaling pathway (Fig. 2D). To explore the immune microenvironment, we analyzed the infiltration of 22 immune cell types using CIBERSORTx. Compared with controls, the PCOS group exhibited increased levels of neutrophils, while gamma delta T cell infiltration decreased. Notably, various activated immune cell types were elevated in PCOS, which may be associated with the inflammatory state (Fig. 2E, F).

### Screening for hub genes by WGCNA

**Fig. 3:**
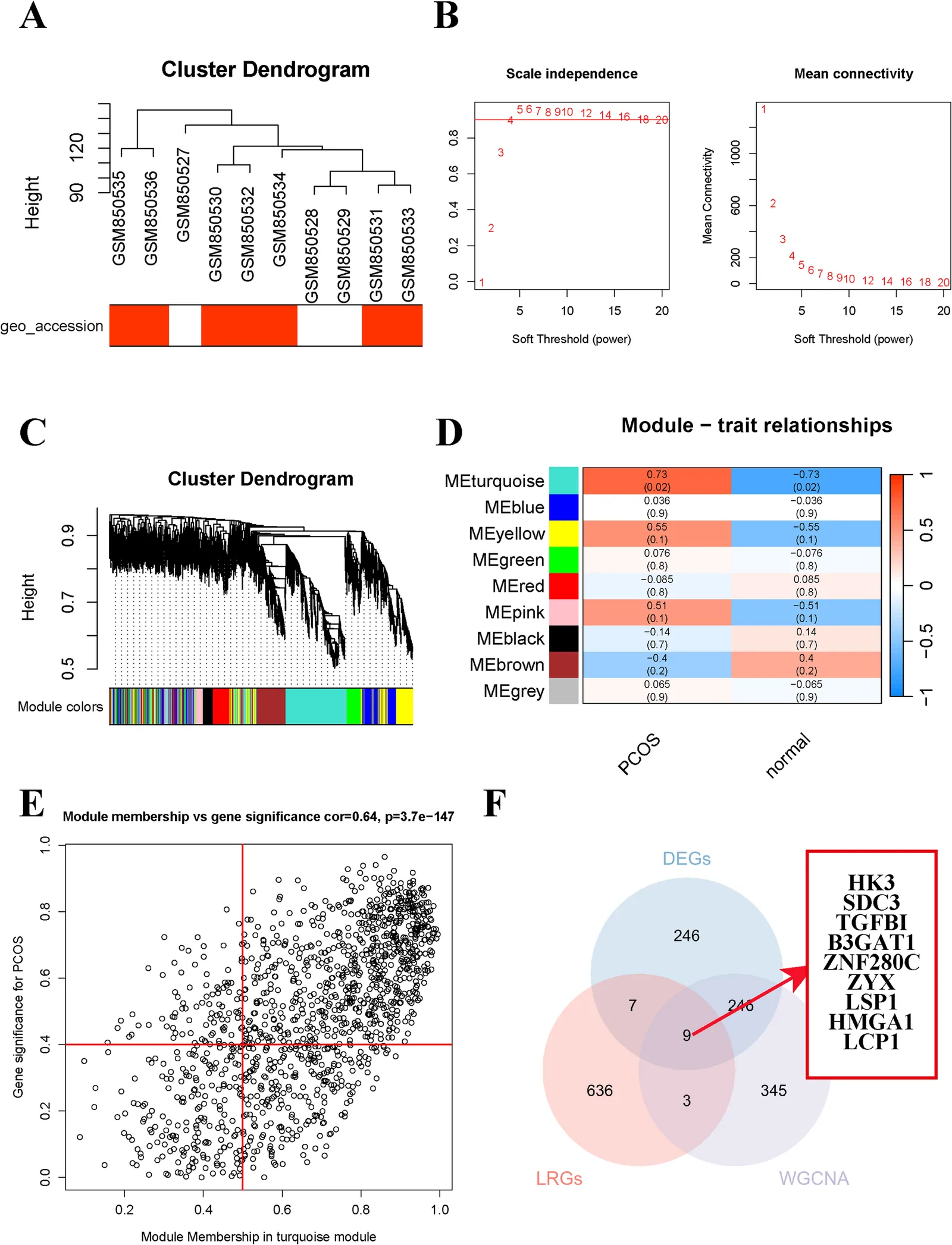
Construction and module analysis of WGCNA. **A** Cluster dendrogram of gene expression profiles. **B** Determination of soft-thresholding power for PCOS. **C **Cluster dendrogram of PCOS highly connected genes in key modules. **D** Correlation between co-expressed gene modules and clinical traits in PCOS; the MEturquoise module shows a positive correlation with PCOS. **E** Genes in the MEturquoise module exhibiting correlation coefficient > 0.5. **F** Identification of nine LRGs significantly associated with PCOS as candidate hub genes

To further identify hub genes most strongly associated with PCOS, we performed WGCNA on cohorts of PCOS patients and healthy individuals. Based on the approximate scale-free topology criterion, we set the scale–free topology fitting index (β) to 4 (Fig. 3A, B). Subsequently, the Topological Overlap Matrix (TOM) was used to detect gene modules correlated with clinical features. Nine gene modules were identified in the PCOS model after merging similar modules. The MEturquoise module exhibited the strongest positive correlation with PCOS (*R* = 0.73, *P* = 0.02), and contained 1268 genes (Fig. 3C, D). From the MEturquoise module, 603 genes with a correlation coefficient > 0.5 were selected based on their significance. The intersection of these genes with the previously identified DEGs and LRGs yielded nine candidate hub genes: HK3, SDC3, TGFBI, B3GAT1, ZNF280C, ZYX, LSP1, HMGA1, and LCP1 (Fig. 3E, F). Detailed information on these hub genes is presented in Table 2.

**Table 2:** Hub genes and their functions

Gene names	Description	Biological function
HK3	Hexokinase 3	Catalyzes the phosphorylation of hexose, such as D-glucose and D-fructose, to hexose 6-phosphate.
SDC3	Syndecan 3	Cell surface proteoglycan that may bear heparan sulfate.
TGFBI	Transforming Growth Factor – Beta-Induced	Cell adhesion. Cell-collagen interactions
B3GAT1	Beta-1,3-Glucuronyltransferase 1	Involved in the biosynthesis of L2/HNK-1 carbohydrate epitope on glycoproteins. Glycosaminoglycan biosynthesis.
ZNF280C	Zinc Finger Protein 280C	Transcription factor.
ZYX	Zyxin	Adhesion plaque protein. Binds alpha-actinin and the CRP protein.
LSP1	Lymphocyte Specific Protein 1	Mediating neutrophil activation and chemotaxis.
HMGA1	High Mobility Group AT-Hook 1	HMG–I/Y bind preferentially to the minor groove of A + T rich regions in double-stranded DNA. Involved in the transcription regulation of genes containing, or in close proximity to A + T-rich regions.
LCP1	Lymphocyte Cytosolic Protein 1	Actin–binding protein. Activation of T-cells in response to costimulation through TCR/CD3 and CD2 or CD28. Modulates the cell surface expression of IL2RA/CD25 and CD69

### Gene Set Enrichment Analysis (GSEA) and Transcription Factor (TF) network analysis of hub genes

**Fig. 4:**
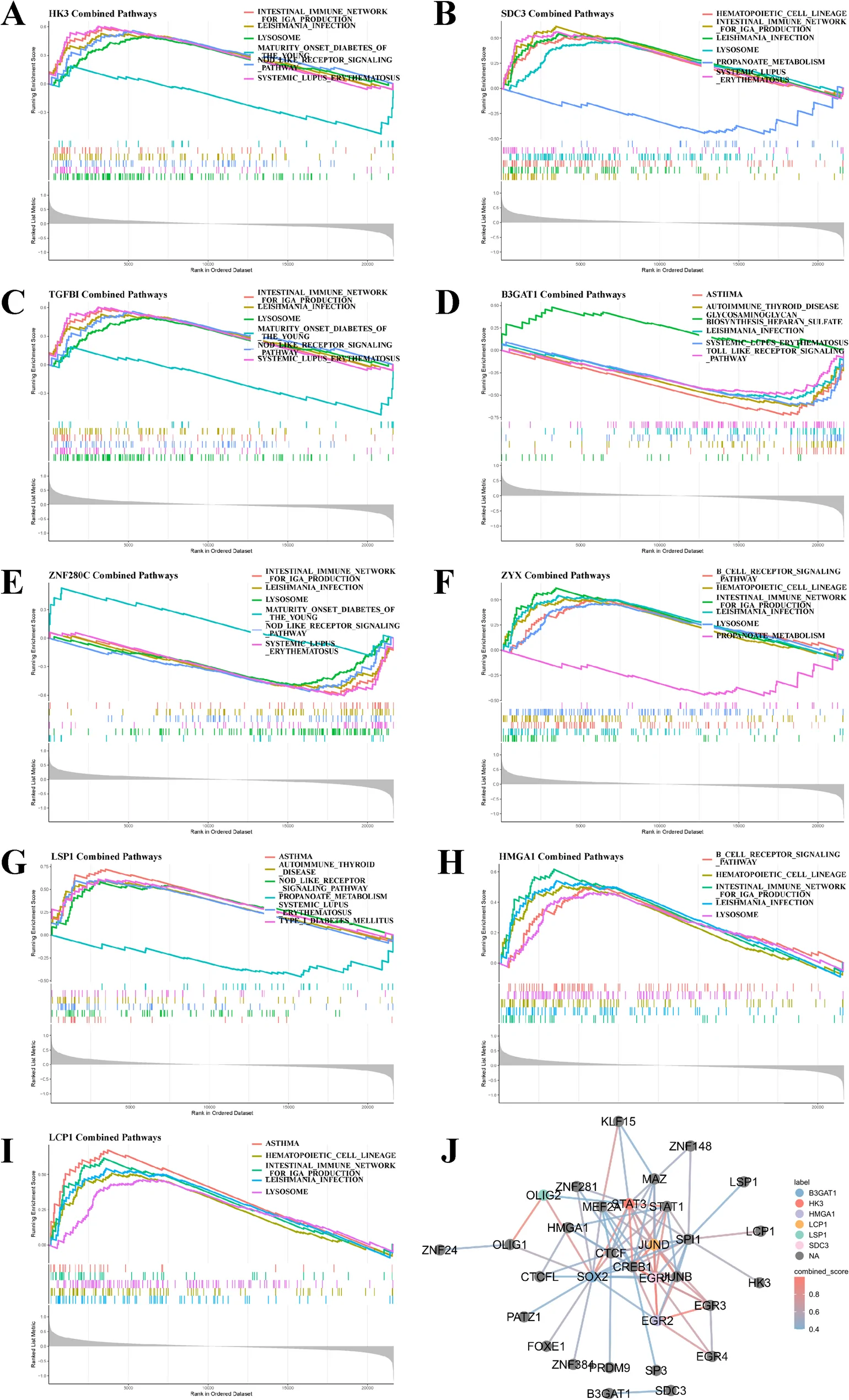
GSEA and TF network analysis of hub genes. **A–I**. GSEA pathway results for nine genes associated with PCOS. **J**. Illustrates the transcription factor network analysis for the nine genes associated with PCOS

To elucidate the functional roles and regulatory mechanisms of the nine hub genes in PCOS, we performed GSEA and TF network analysis. GSEA results revealed distinct expression patterns: only B3GAT1 and ZNF280C were downregulated, while the remaining genes were upregulated in PCOS. These genes were mainly positively enriched in multiple immune-related signaling pathways, including the intestinal immune network for IgA production, leishmania infection, lysosome, NLRs signaling pathway, hematopoietic cell lineage, systemic lupus erythematosus, asthma, autoimmune thy, TLRs signaling pathway, B cell receptor signaling pathway, and type I diabetes mellitus. Conversely, they were negatively associated with metabolic pathways such as maturity–onset diabetes of the young, propanoate metabolism, and glycosaminoglycan biosynthesis heparan–sulfate (Fig. 4A–I; Table 3). Furthermore, TF network analysis identified JUND, STAT3, EGR2, EGR1, and OLIG2 as potential core transcription factors (Fig. 4J).

**Table 3:** Analysis of hub genes and their enrichment in signaling pathways

Gene	PCOS vs. WT	Upregulated	Downregulated
HK3	UP	KEGG_INTESTINAL_IMMUNE_NETWORK_FOR_IGA_PRODUCTION KEGG_LEISHMANIA_INFECTION KEGG_LYSOSOME KEGG_NOD_LIKE_RECEPTOR_SIGNALING_PATHWAY	KEGG_MATURITY_ONSET_DIABETES_OF_THE_YOUNG
SDC3	UP	KEGG_HEMATOPOIETIC_CELL_LINEAGE KEGG_INTESTINAL_IMMUNE_NETWORK_FOR_IGA_PRODUCTION KEGG_LEISHMANIA_INFECTION KEGG_LYSOSOME KEGG_SYSTEMIC_LUPUS_ERYTHEMATOSUS	KEGG_PROPANOATE_METABOLISM
TGFBI	UP	KEGG_INTESTINAL_IMMUNE_NETWORK_FOR_IGA_PRODUCTION KEGG_LEISHMANIA_INFECTION KEGG_LYSOSOME KEGG_NOD_LIKE_RECEPTOR_SIGNALING_PATHWAY KEGG_SYSTEMIC_LUPUS_ERYTHEMATOSUS	KEGG_MATURITY_ONSET_DIABETES_OF_THE_YOUNG
B3GAT1	DOWN	KEGG_GLYCOSAMINOGLYCAN_BIOSYNTHESIS_HEPARAN_SULFATE	KEGG_ASTHMA KEGG_AUTOIMMUNE_THYROID_DISEASE KEGG_LEISHMANIA_INFECTION KEGG_SYSTEMIC_LUPUS_ERYTHEMATOSUS KEGG_TOLL_LIKE_RECEPTOR_SIGNALING_PATHWAY
ZNF280C	DOWN	KEGG_MATURITY_ONSET_DIABETES_OF_THE_YOUNG	KEGG_INTESTINAL_IMMUNE_NETWORK_FOR_IGA_PRODUCTION KEGG_LEISHMANIA_INFECTION KEGG_LYSOSOME KEGG_NOD_LIKE_RECEPTOR_SIGNALING_PATHWAY KEGG_SYSTEMIC_LUPUS_ERYTHEMATOSUS
ZYX	UP	KEGG_B_CELL_RECEPTOR_SIGNALING_PATHWAY KEGG_HEMATOPOIETIC_CELL_LINEAGE KEGG_INTESTINAL_IMMUNE_NETWORK_FOR_IGA_PRODUCTION KEGG_LEISHMANIA_INFECTION KEGG_LYSOSOME	KEGG_PROPANOATE_METABOLISM
LSP1	UP	KEGG_ASTHMA KEGG_AUTOIMMUNE_THYROID_DISEASE KEGG_NOD_LIKE_RECEPTOR_SIGNALING_PATHWAY KEGG_SYSTEMIC_LUPUS_ERYTHEMATOSUS KEGG_TYPE_I_DIABETES_MELLITUS	KEGG_PROPANOATE_METABOLISM
HMGA1	UP	KEGG_B_CELL_RECEPTOR_SIGNALING_PATHWAY KEGG_HEMATOPOIETIC_CELL_LINEAGE KEGG_INTESTINAL_IMMUNE_NETWORK_FOR_IGA_PRODUCTION KEGG_LEISHMANIA_INFECTION KEGG_LYSOSOME	–
LCP1	UP	KEGG_ASTHMA KEGG_HEMATOPOIETIC_CELL_LINEAGE KEGG_INTESTINAL_IMMUNE_NETWORK_FOR_IGA_PRODUCTION KEGG_LEISHMANIA_INFECTION KEGG_LYSOSOME	–

### Establishment and functional analysis of subtypes based on hub genes

**Fig. 5:**
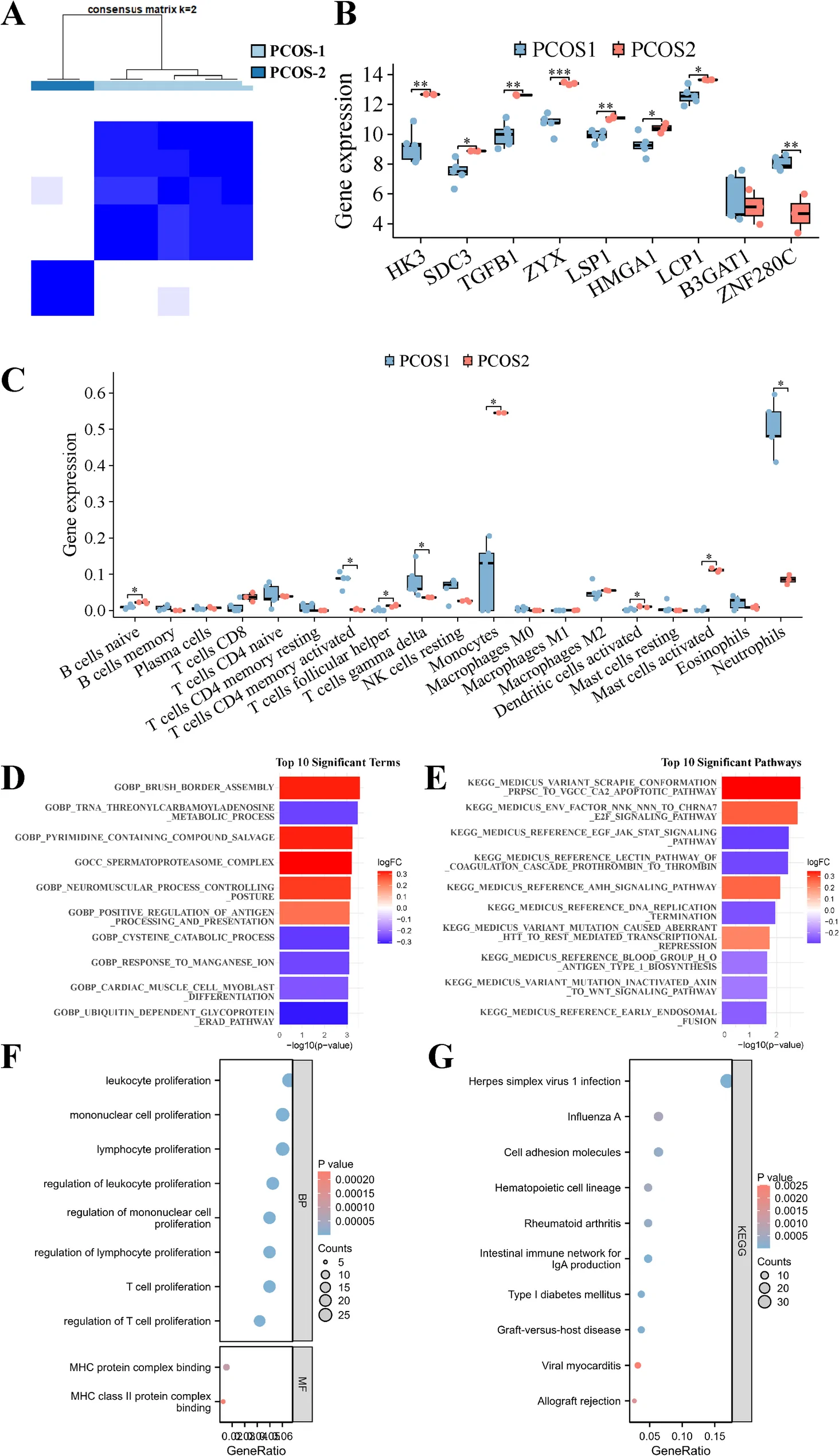
Subtype analysis of PCOS based on hub genes. **A** Consensus clustering identifies two distinct subtypes of PCOS patients. **B** The expression patterns of nine genes are compared between the two subtypes. **C** Comparative analysis of immune cell infiltration levels reveals significant differences between the two subtypes. **D** GSVA analysis of GO terms for the two subtypes. **E** GSVA analysis of KEGG pathways for the two subtypes. **F** GO enrichment analysis of DEGs between the two subtypes. **G** KEGG pathway enrichment analysis of DEGs between the two subtypes. (*p < 0.05)

Unsupervised consensus clustering analysis based on the expression of the nine hub genes successfully divided PCOS patients into two groups: PCOS1 and PCOS2 (Fig. 5A). Differential expression analysis between these subtypes revealed distinct gene profiles. In the PCOS2 subtype, seven hub genes (HK3, SDC3, TGFBI, ZYX, LSP1, HMGA1, and LCP1) were significantly upregulated, while ZNF280C was downregulated; B3GAT1 expression showed no significant difference between the two groups (Fig. 5B). We subsequently analyzed the differences in immune cell infiltration between the two subtypes. Activated CD4 memory T cells, gamma delta T cells and neutrophils were significantly elevated in PCOS1, whereas naive B cells, follicular helper T cells, monocytes, activated dendritic cells and activated mast cells were notably increased in PCOS2 (Fig. 5C). GSVA assessments indicated that the PCOS2 subtype showed heightened activity in pathways related to the spermatoproteasome complex, antigen processing and presentation, apoptosis, E2F targets, and HTT-mediated transcriptional repression (Fig. 5D, E). Conversely, the PCOS1 subtype displayed greater activity in metabolic processes such as ubiquitin-dependent protein degradation, as well as in the EGF-JAK-STAT and AXIN-Wnt signaling pathways (Fig. 5D, E). To further analyze differences between PCOS1 and PCOS2, we identified 522 DEGs between PCOS1 and PCOS2 (|log_2_FC| > 2, adjusted *p*-value < 0.05). Functional enrichment analysis of these DEGs highlighted significant immune involvement. GO analysis revealed enrichment in biological processes (BP) regulating immune cell proliferation, including leukocytes, mononuclear lymphocytes and T cells, as well as molecular functions (MF) linked to MHC and MHC class II protein complex binding (Fig. 5F). KEGG pathway analysis similarly indicated significant enrichment in cell adhesion molecules, the intestinal immune network for IgA production, and the inflammatory response pathways (Fig. 5G).

### Construction and validation of diagnostic model by machine learning

**Fig. 6:**
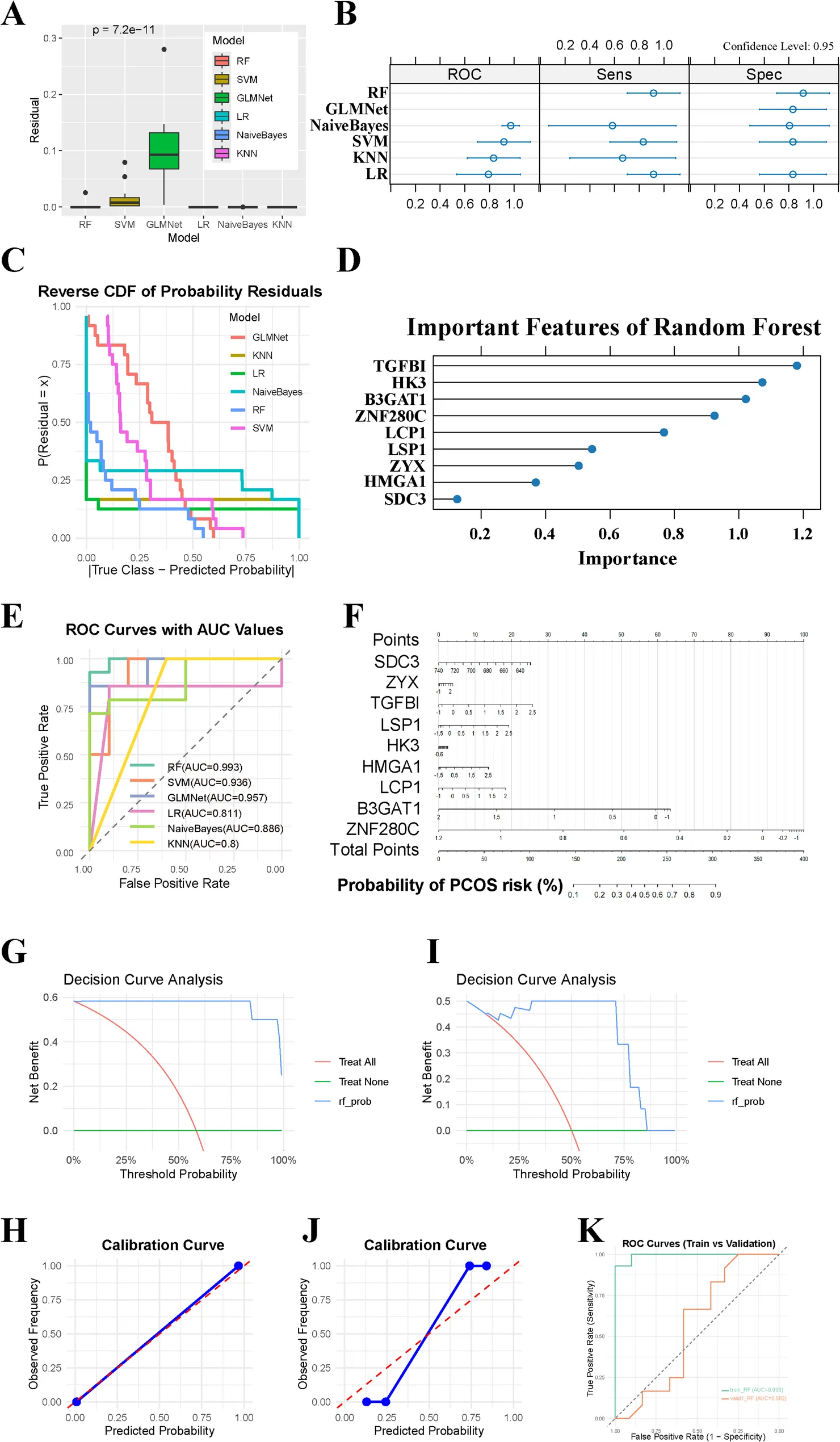
Machine learning analysis of PCOS based on lactylation-related transcriptional signatures. **A** Comparison of several machine learning models, including the RF, SVM, GLM, LR, NB, and KNN. Boxplots of residuals demonstrate that the RF model has lower residual values. **B** A chart illustrates the performance comparison of six distinct machine learning algorithms. The points represent the performance values of the algorithm under the corresponding metric, and the error bars reflect the confidence interval at a confidence level of 0.95. **C** Residual distribution of six models were shown through the reverse cumulative distribution of their residuals. **D** The important features in the random forest model. **E** ROC analysis of the seven models. **F** Nomogram used for predicting PCOS risk. **G** The red line, representing the model based on the genes in the decision curve analysis, deviates from the gray and black lines, indicating the feasibility of the model. **H **Calibration and decision curve analyses were used to evaluate the exploratory predictive performance of the model. **I–K**. Validation results from an external validation dataset

To evaluate the diagnostic potential of the nine hub genes (HK3, SDC3, TGFBI, B3GAT1, ZNF280C, ZYX, LSP1, HMGA1, and LCP1) for PCOS, we employed six machine learning algorithms: Random Forest (RF), Support Vector Machine (SVM), Generalized Linear Model (GLM), Logistic Regression (LR), Naïve Bayes (NB), and K–Nearest Neighbors (KNN). Comparative analysis based on residual plots, reverse cumulative distribution of residuals, and ROC curves indicated that the RF algorithm outperformed other methods. RF demonstrated higher overall accuracy, lower residual values, and a larger area under the ROC curve (Fig. 6A–C, E). This performance advantage likely stems from RF’s ability to reduce model variance, handle high–dimensional data effectively, and capture complex nonlinear relationships through ensemble learning, thereby offering greater robustness against noise in smaller datasets. We ranked the nine hub genes by their feature importance in the RF model. TGFBI emerged as the most critical predictor, with the highest importance value (approximately 1.2). The remaining genes, in descending order of importance, were HK3, B3GAT1, ZNF280C, ZYX, LSP1, HMGA1, and LCP1, while SDC3 had the lowest importance value (around 0.2) (Fig. 6D). In the internal dataset, the RF model achieved a high AUC of 0.993, suggesting that the nine-gene signature had strong discriminatory capacity within the discovery cohort (Fig. 6F). This tool assigns individual scores to each gene, and a summed total score estimates the probability of PCOS. Decision curves and calibration curves demonstrated its accuracy (Fig. 6G, H). Finally, we performed rigorous external validation using the independent dataset GSE173160 (Fig. 6I, J). However, the AUC of the independent external dataset decreased to 0.628 (Fig. 6K). This reduction indicates limited external generalizability of the model. The discrepancy between the internal and external AUC values may be related to the small sample size, cohort-specific expression patterns, platform differences between microarray and RNA-seq datasets, batch effects, and biological heterogeneity among PCOS patients. Therefore, the current model should be regarded as a preliminary and hypothesis-generating diagnostic model rather than a clinically validated classifier.

### Expression validation of hub genes and potential therapeutic drugs prediction

**Fig. 7:**
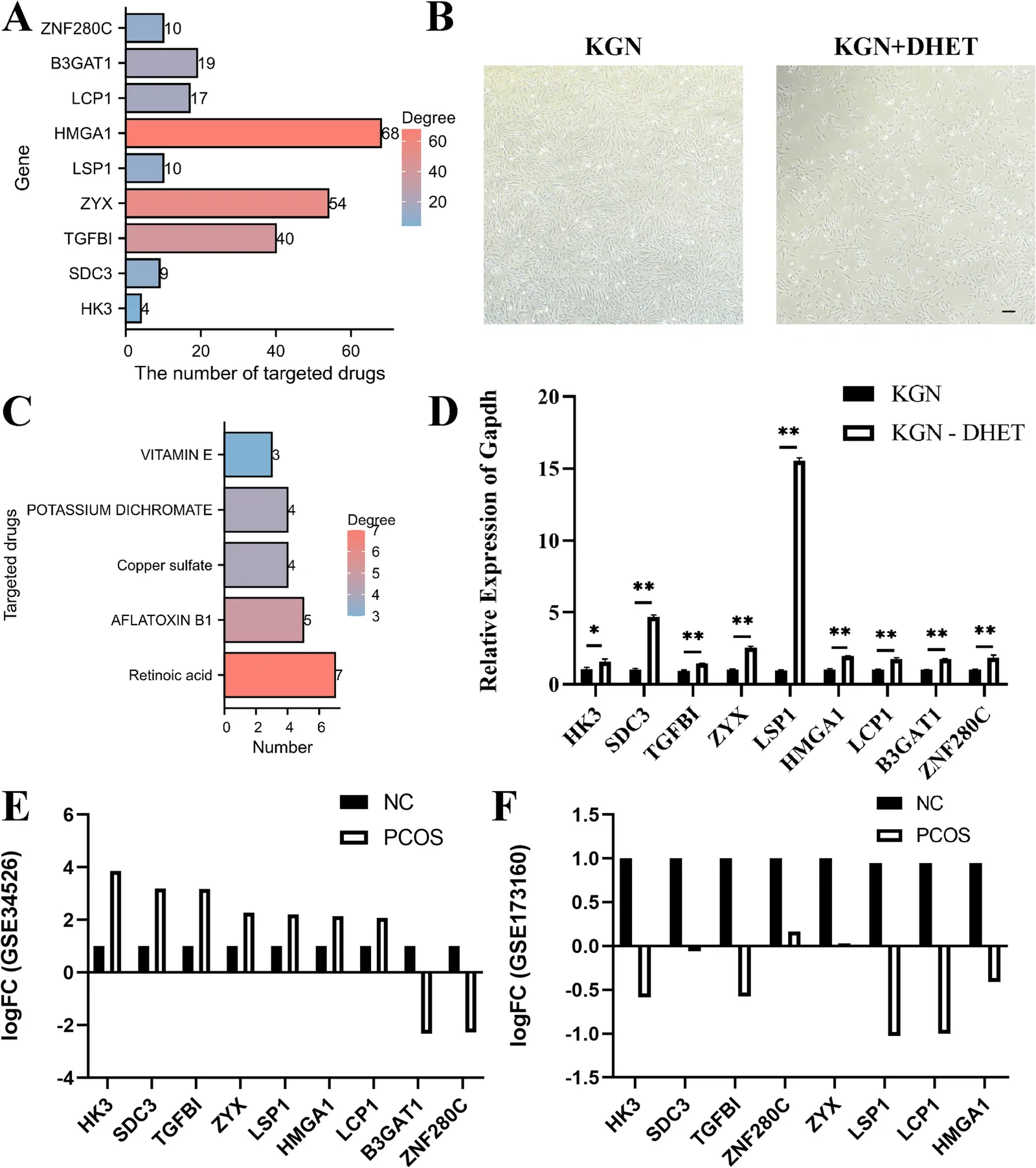
Experimental validation of hub candidate gene expression and prediction of potential therapeutic agents for PCOS. **A** Prediction of hub gene-targeting candidate compounds using the CMap database. Bar chart showing the number of compounds associated with each individual hub gene. **B** Gene set enrichment analysis of the top five candidate compounds in **A**. Retinoic acid-related gene sets were significantly enriched for seven of the nine hub genes. **C** Morphological characteristics of KGN cells following 24 h DHEA treatment. **D** Expression level of hub genes in KGN cells treated with DHEA (*n* = 3; *p < 0.05; **p < 0.01). **E**–**F **Expression level of hub genes in GSE34526 (**E**) and GSE173160 (**F**)

To bridge the gap between our computational findings and experimental validation, and to explore therapeutic implications, we performed a multi–step analysis. First, we utilized the Connectivity Map (CMap) database to predict potential compounds that might reverse the expression signature of the nine hub genes. Figure 7A illustrates the number of compounds predicted to interact with each individual hub gene. From this analysis, we identified the top five candidate compounds with the strongest predicted activity. Subsequent gene set enrichment analysis of these top compounds revealed that retinoic acid was significantly enriched for seven of the nine hub genes (Fig. 7B), suggesting its potential to modulate the lactylation-related gene network implicated in PCOS. To experimentally validate the hub genes and assess the cellular effects of a PCOS-like environment, we employed a human granulosa–like KGN cell line. Cells were treated with 10 µM dehydroepiandrosterone (DHEA) for 24 h to mimic hyperandrogenism, a key feature of PCOS. Morphological observations indicated that DHEA treatment adversely affected cell growth, with evidence of increased cell death or apoptosis compared to the control group (Fig. 7C). In DHEA-treated KGN cells, all nine candidate genes were significantly upregulated compared with controls (Fig. 7D). However, this expression pattern was not fully consistent with the transcriptomic datasets (Fig. 7E, F), in which B3GAT1 and ZNF280C showed decreased expression in PCOS samples. This discrepancy may be attributable to differences between tumor-derived KGN cells and primary granulosa cells, the acute hyperandrogenic stimulation model, and the biological heterogeneity of human PCOS tissues. Therefore, the in vitro results should be interpreted as preliminary evidence that these genes respond to a PCOS-like hyperandrogenic condition, rather than direct confirmation of the patient-derived expression patterns. Taken together, these results not only provide strong experimental support for the involvement of the identified LRGs in PCOS pathophysiology but also identify retinoic acid as a promising candidate therapeutic agent warranting further investigation for PCOS treatment.

## Discussion

Polycystic ovary syndrome (PCOS) is a prevalent endocrine disorder affecting approximately 10–13% of women of reproductive age. It is characterized by a combination of clinical features, including hyperandrogenism, ovulatory dysfunction, and polycystic ovarian morphology, leading to significant reproductive and metabolic complications such as infertility and an elevated risk of metabolic syndrome [1]. The current diagnosis, primarily based on the Rotterdam criteria [31], relies on clinical and ultrasonographic features, which can be subjective and may delay intervention. Consequently, there is an urgent need to identify novel, objective biomarkers for early and precise diagnosis to improve patient management and outcomes [4, 32, 33] .

Building upon prior transcriptomic and systems biology research in PCOS [6–8], this study aimed to investigate whether lactylation–related transcriptional signatures could serve as potential novel biomarkers for PCOS. Through integrated bioinformatics analyses, we identified nine core LRGs—HK3, SDC3, TGFBI, ZYX, LSP1, HMGA1, LCP1, B3GAT1, and ZNF280C—that were significantly dysregulated in PCOS granulosa cells (Fig. 3F). The association of most of these genes with PCOS has been rarely reported, underscoring their potential as novel diagnostic markers. The role of TGFBI in PCOS appears context-dependent [34]. Rodgers et al. [35] and Zhang et al. [36] all reported decreased TGFBI expression in fetal ovarian fibroblasts, which was linked to altered expression of several PCOS candidate genes, suggesting a role in developmental susceptibility. In contrast, our study identified TGFBI upregulation in adult PCOS ovarian tissues, which is consistent with the results of recently published studies [34]. This discrepancy may reflect dynamic, stage-specific changes in TGFBI function during ovarian development and PCOS pathogenesis, warranting further mechanistic investigation.

Our finding of elevated HMGA1 expression aligns with previous reports [37]. As a key transcriptional regulator of the insulin receptor (INSR) gene, HMGA1 is intricately linked to insulin resistance, a core pathological feature of PCOS [38, 39]. Furthermore, Zhu et al. [40] identified that HMGA1 is a key transcription factor among ferroptosis-related genes in granulosa cells (GCs) of PCOS patients, reinforcing its significance as a promising biomarker for early diagnosis.

Pathway enrichment analysis suggested that these candidate genes are involved in immune-inflammatory pathways (IgA production, TLR/NLR signaling, and autoimmune diseases) and highlight metabolic-immune crosstalk (type I diabetes, hematopoietic lineage) (Fig. 4A–I). This is consistent with the established concept of chronic low-grade inflammation in PCOS pathogenesis [41–43]. For instance, Billig et al. [44, 45] found that HMGB1, via binding to TLR2/TLR4, activates the NFκB signaling pathway to promote cellular inflammatory responses, leading to PCOS. Moreover, Escobar-Morreale et al. [46] reported that genetic polymorphisms in TLR2 are associated with PCOS susceptibility. Our research also predicts two novel regulatory mechanisms underlying PCOS: (1) Enrichment in the Leishmania infection pathway suggests a potential pathogen-immune interaction that may exacerbate the Th17/Treg imbalance observed in PCOS, analogous to mechanisms in other chronic inflammatory conditions [43, 47, 48]. (2) Enrichment in lysosomal pathways points to possible dysfunction in organelle clearance; impaired lysosomal activity could lead to defective mitophagy, resulting in reactive oxygen species (ROS) accumulation and accelerated granulosa cell senescence, thereby contributing to PCOS pathophysiology [49, 50]. However, we interpret these enrichment results with caution. They represent transcriptomic associations within the predefined LRG set and do not establish lactylation as the direct driver of these pathways.

Interestingly, while overall immune cell infiltration showed few significant differences between PCOS patients and healthy controls (Fig. 2E, F), a more nuanced pattern emerged after stratification. Unsupervised clustering based on hub gene expression identified two distinct molecular subtypes of PCOS (PCOS1 and PCOS2). These subtypes exhibited markedly different immune profiles: PCOS1 displayed enrichment for activated CD4 memory T cells, γδ T cells, and neutrophils, whereas PCOS2 displayed enrichment for naive B cells, follicular helper T cells, monocytes, and activated dendritic/mast cells (Fig. 5C). This suggests that LRGs not only serve as diagnostic tools but may also define immunologically distinct PCOS endotypes, potentially guiding personalized therapeutic strategies aimed at restoring immune homeostasis. However, given the small sample size, conclusions regarding specific immune cell populations (e.g., from CIBERSORT analysis) are preliminary and require validation in larger cohorts with direct immunological measurements.

Although the RF model achieved high internal performance, the external validation performance was modest, with an AUC of 0.628. This discrepancy suggests that the high internal AUC may partly reflect overfitting to the discovery cohort. Several factors may have contributed to the reduced external performance, including the very small sample size, differences between microarray and RNA-seq platforms, batch effects, clinical heterogeneity of PCOS, and instability of feature selection in high-dimensional transcriptomic data. In addition, because the candidate genes were selected using the discovery dataset before model construction, potential feature-selection bias could not be fully excluded. Therefore, the proposed nine-gene signature should be interpreted as an exploratory biomarker panel, and its clinical applicability requires validation in larger, multicenter, prospective cohorts.

The in vitro validation using DHEA-treated KGN cells provided preliminary support for the androgen-responsiveness of some candidate genes. However, several critical limitations must be emphasized. First, KGN cells are tumor-derived and may not fully recapitulate the physiology of primary PCOS granulosa cells [51, 52]. Second, the DHEA model primarily mimics hyperandrogenism, only one aspect of PCOS heterogeneity. Most importantly, we observed a notable inconsistency: while patient transcriptomic data showed mixed up- and down-regulation (e.g., B3GAT1 and ZNF280C were down), all tested genes were uniformly upregulated in the DHEA-treated KGN model (Fig. 7E). This divergence underscores the substantial gap between simplified in vitro models and the complex in vivo milieu of PCOS, highlighting that the cell line results are supportive but not definitive confirmations of the human data.

This study has several key limitations. Primarily, it is based on transcriptomic inference from publicly available datasets and a predefined LRG set. It does not provide direct evidence of protein lactylation, enzymatic activity, or causal mechanisms. The biomarker model is exploratory and requires rigorous external validation. The in vitro model has inherent constraints as noted. Finally, the precise biological role and regulatory impact of lactylation on the identified genes in PCOS pathogenesis remain to be elucidated through direct lactylation assays and functional experiments [53].

## Conclusion

In summary, this exploratory study identified a nine-gene lactylation-related transcriptional signature that is dysregulated in PCOS and associated with immune-metabolic pathways. This signature also revealed potential molecular subtypes, offering a novel lens for patient stratification. Importantly, our findings are derived from transcriptomic inference and a predefined gene set, without direct validation of lactylation. The diagnostic model showed limited generalizability, underscoring the hypothesis-generating nature of this work. Therefore, this signature serves as a set of candidate biomarkers and a foundation for future research. Subsequent studies must prioritize validation in large cohorts, functional assays in primary cells, and direct measurement of lactylation to translate these observations into mechanistic insight. 

## Supplementary Information

Supplementary Material 1.

Supplementary Material 2.

Supplementary Material 3.

Supplementary Material 4.

## Data Availability

The data from RNA-seq will be shared upon reasonable request to the corresponding author. The publicly available dataset used in this study can be downloaded from the GEO database (http://www.ncbi.nlm.nih.gov/geo/).

## Abbreviations

PCOS: Polycystic ovary syndrome
GEO: the Gene Expression Omnibus
LRG: Lactylation-related gene
DEGs: Differentially expressed genes
GO: Gene Ontology
KEGG: Kyoto encyclopedia of genes and genomes
WGCNA: Weighted gene co-expression network analysis
TOM: Topological Overlap Matrix
GSEA: Gene Set Enrichment Analysis
TFs: Transcription factors
PPI: Protein-protein interaction
GSVA: Gene Set Variation Analysis
RF: Random Forest
SVM: Support Vector Machine
GLM: Generalized Linear Model
LR: Logistic Regression
NB: Naive Bayes
KNN: K-Nearest Neighbors
ROC: Receiver operating characteristic
DCA: Decision curve analysis
DHEA: Dehydroepiandrosterone
DMSO: Dimethyl Sulfoxide
qRT–PCR: Quantitative real-time reverse transcription polymerase chain reaction
BP: Biological processes
MF: Molecular functions
GCs: Granulosa cells
